# Household Composition and Inequalities in COVID-19 Vaccination in Wales, UK

**DOI:** 10.3390/vaccines11030604

**Published:** 2023-03-07

**Authors:** Alex Lench, Malorie Perry, Rhodri D. Johnson, Richard Fry, Gill Richardson, Ronan A. Lyons, Ashley Akbari, Adrian Edwards, Brendan Collins, Natalie Joseph-Williams, Alison Cooper, Simon Cottrell

**Affiliations:** 1 Vaccine Preventable Disease Programme and Communicable Disease Surveillance Centre, Public Health Wales, 2 Capital Quarter, Tyndall Street, Cardiff CF10 4BZ, UK; 2 Population Data Science, Health Data Research UK, Swansea University Medical School, Swansea SA2 8PP, UK; 3 Policy, Research and International Development, Public Health Wales, 2 Capital Quarter, Tyndall Street, Cardiff CF10 4BZ, UK; 4 Wales COVID-19 Evidence Centre, PRIME Centre Wales, Division of Population Medicine, School of Medicine, Cardiff University, 8th floor, Neuadd Meirionnydd, Heath Park, Cardiff CF14 4XN, UK; 5 Health and Social Services Group, Finance Directorate, Welsh Government, Cardiff CF10 3NQ, UK

**Keywords:** COVID-19, vaccines, vaccination, immunisation, households, household composition, inequities, inequalities

## Abstract

The uptake of COVID-19 vaccination in Wales is high at a population level but many inequalities exist. Household composition may be an important factor in COVID-19 vaccination uptake due to the practical, social, and psychological implications associated with different living arrangements. In this study, the role of household composition in the uptake of COVID-19 vaccination in Wales was examined with the aim of identifying areas for intervention to address inequalities. Records within the Wales Immunisation System (WIS) COVID-19 vaccination register were linked to the Welsh Demographic Service Dataset (WDSD; a population register for Wales) held within the Secure Anonymised Information Linkage (SAIL) databank. Eight household types were defined based on household size, the presence or absence of children, and the presence of single or multiple generations. Uptake of the second dose of any COVID-19 vaccine was analysed using logistic regression. Gender, age group, health board, rural/urban residential classification, ethnic group, and deprivation quintile were included as covariates for multivariable regression. Compared to two-adult households, all other household types were associated with lower uptake. The most significantly reduced uptake was observed for large, multigenerational, adult group households (aOR 0.45, 95%CI 0.43–0.46). Comparing multivariable regression with and without incorporation of household composition as a variable produced significant differences in odds of vaccination for health board, age group, and ethnic group categories. These results indicate that household composition is an important factor for the uptake of COVID-19 vaccination and consideration of differences in household composition is necessary to mitigate vaccination inequalities.

## 1. Introduction

The COVID-19 pandemic was characterised by diverse inequalities and following the development of vaccines, this included widespread differences in the uptake of immunisation. Previous work by this group in 2021 characterised a number of predictors for COVID-19 vaccine uptake [1]. This study, and others, identified multiple inequalities in COVID-19 vaccination with differences by ethnic group being amongst the most severe [1,2]. Whilst previous research has demonstrated inequalities in COVID-19 vaccine uptake, they have also indicated that further work was necessary to characterise patterns of coverage and provide insights for potential interventions to address the inequalities identified.

The role of housing as an important factor in health and wellbeing is well established and the characterisation of households in terms of the number and relative characteristics of the residents (household composition) is a key consideration in this area [3]. In particular, isolation is well understood as a risk factor for a broad range of adverse health and social issues [4], though other considerations relating to household composition, such as overcrowding and multi-generational living are also important and can be associated with factors such as ethnic group [5,6].

Methodological advances developed by Rodgers et al. [7] have allowed anonymous individual-level data to be linked using unique residence identifiers within the Secure Anonymised Information Linkage (SAIL) databank in Wales [8]. While this technique cannot currently provide information on the type of relationships between different residents of a property, it can be used to effectively characterise households by household size, age of residents, and generational composition [9]. In the present study, we aimed to use this methodology to examine the influence of household composition on COVID-19 vaccination coverage and inequalities.

## 2. Materials and Methods

Analyses were completed within the SAIL Databank, hosted by Swansea University, as part of the Con-COV (Controlling COVID-19) project [10,11], using the software package R (version 4.1.3). Con-COV is a total population electronic cohort derived from linking many databases [10]. All individuals alive and resident in Wales on 1 January 2022 were included in the initial cohort, identified from the Welsh Demographic Service dataset (WDSD). In Wales, vaccination for COVID-19 began on 8 December 2020, and delivery of first and second doses in the adult population plateaued by one year.

Vaccination status, sex, urban/rural location of residence, health board of residence, ethnic group, and deprivation quintile of Lower-Layer Super Output Area were assigned as previously described [1]. Ethnic group categories were defined by the Office for National Statistics 2011 Census [12]. Individuals with learning disabilities were identified using standard guidance on clinical coding for identifying vaccine eligibility, monitoring/reporting of vaccine uptake, and call/recall purposes, provided by the University of Nottingham PRIMIS group [13].

Household size and generational composition were calculated using an encrypted Unique Property Reference Number, known as a Residential Anonymised Linkage Field (RALF), as previously described [7,9]. Generational composition was calculated using the ‘Relative Age to Youngest’ (AtY) method [9]. Where the age between the oldest and youngest people in the household is 18 years or less, the household was classified as having one generation and where the age between the oldest and youngest people in the household is over 18 years, the household was classified as having multiple generations.

Mutually exclusive household composition groups (referred to as household types) were calculated based on household size, generational composition, and the presence/absence of at least one child (person < 18 years old) in the household as described in Table 1. Household types were sole occupancy (1), two adults (2), adults/children (3), large adults/children (4), adult group single/multiple generations (5/6), and large adult group single/multiple generations (7/8).

Adults living in households of size 10 or above were excluded from the analysis as these households are likely to contain a substantial proportion of communal residences, such as care homes or supported living where immunisation is coordinated and monitored directly via the residence. Persons with immunocompromised status were also excluded due to this (relatively small) group having a three-dose primary course.

Vaccination of adults (aged 18 years or over as of 31 March 2022) with a complete primary course (i.e., first two doses) of any COVID-19 vaccine, received as of 1 January 2022, was analysed. Uptake figures were calculated overall and stratified by sex, age group, rural/urban classification, learning disability, and ethnic group. The odds of vaccination with a complete primary course were estimated using univariable and multivariable logistic regression. Forward step-wise selection, based on optimising the Akaike information criterion value and consideration of likely confounders, was used to select the adjustment variables for the multivariable model with sex, age group, rural/urban classification, health board of residence, ethnic group, and deprivation quintile included in the analysis. The potential influence of correlations within households was assessed by duplication of analyses using cluster-robust regression with RALF assigned as a clustered factor but no significant differences were detected [14].

## 3. Results

**Figure 1:**
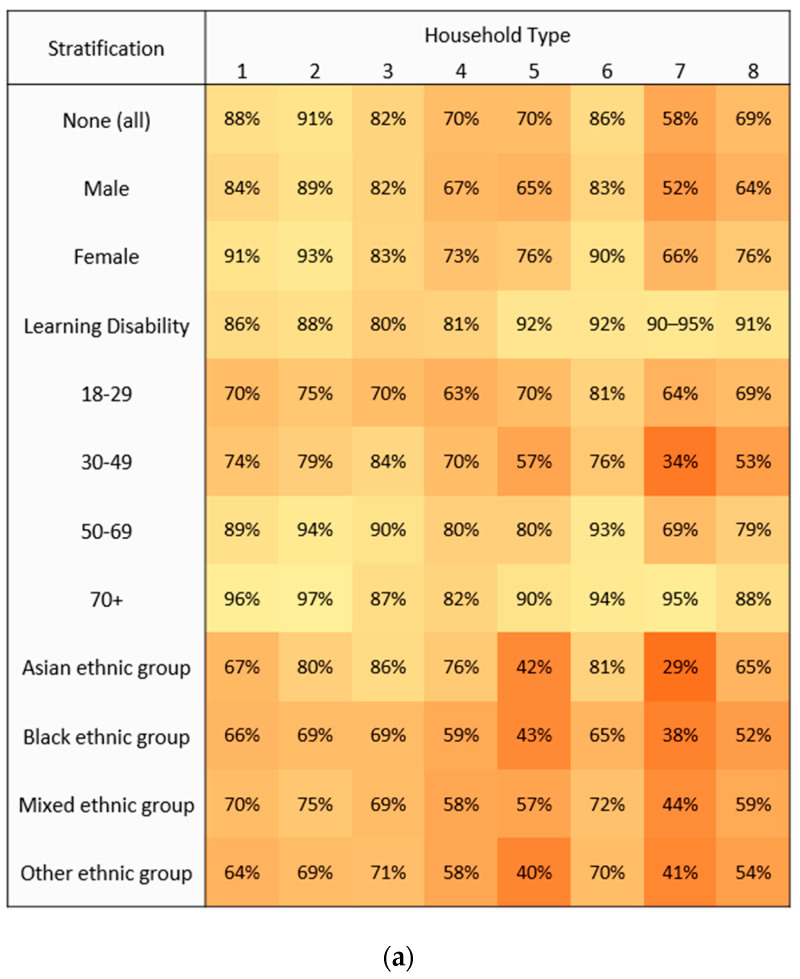

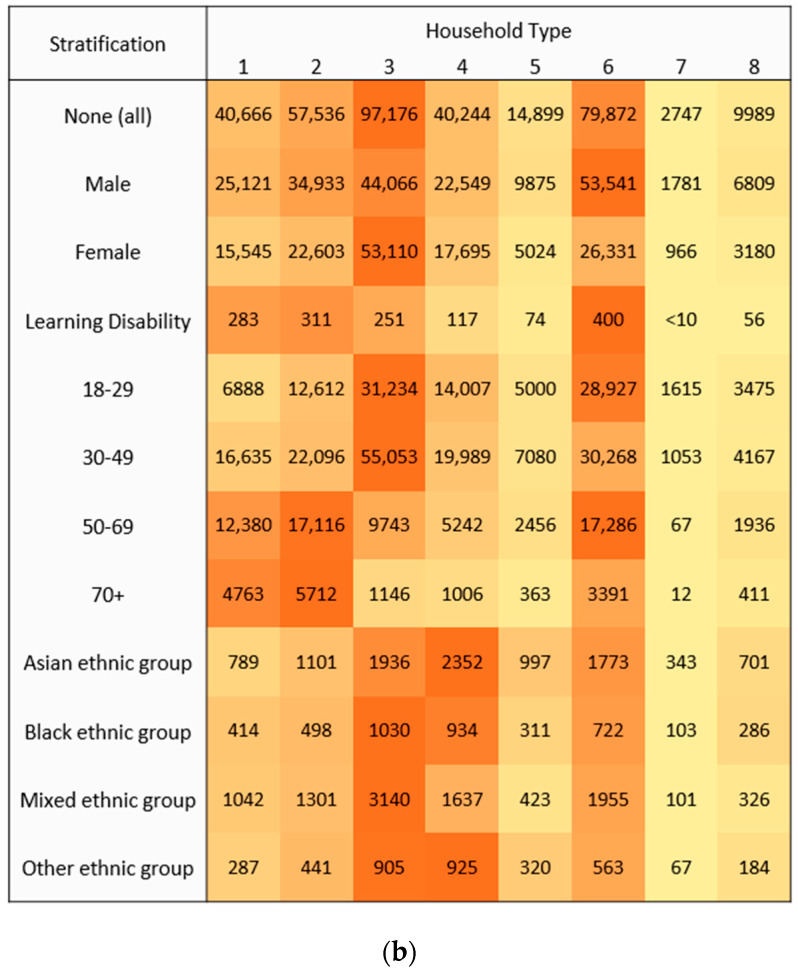
Vaccination with a complete two-dose course of COVID-19 vaccine (any type) by household type, stratified for sex, learning disability, age group, and Asian, Black, Mixed, and Other ethnic groups, Wales 2020–2021. (**a**) Heat map for percentage uptake with light-dark shading for higher to lower percentages across stratified categories. (**b**) Heat map for number of non-vaccinated people with light-dark shading for lower to higher numbers within stratified categories. Data sourced from the all Wales Immunisation System (WIS) in SAIL, COVID-19 Vaccination Data (CVVD), as of 1st January 2022. Ranges are given where necessary to mask low numbers.

The study population included 2,317,275 individuals aged 18 years or older and alive and resident in Wales on the 1st of January 2022. Differences in uptake for two doses of the COVID-19 vaccine were observed across household types when calculated overall and for multiple subpopulations (Figure 1). Denominator figures are shown in Supplementary Table S2. The highest percentage uptake was observed for people aged 70 and over living in two-adult households (97%, *n* = 213,092), and the lowest percentage uptake was for people from an Asian ethnic group living in large adult group, single-generation households (29%, *n* = 482).

The highest number of unvaccinated people overall were in adult/children households (97,176) and multi-generation adult groups (79,872). For people with learning disabilities, the largest number of unvaccinated people lived in multi-generation adult groups (400) and two-adult (311) households. For those aged 70 years and older, the highest numbers of unvaccinated people were in single occupancy (4763) and two-adult (5712) household types. Asian, Black, and Other ethnic group categories had the highest numbers of unvaccinated people in adults/children (1936, 1030, and 905, respectively) and large adults/children (2352, 934, and 925, respectively) household types.

In a univariable analysis, the odds of having received a complete primary course were significantly less for individuals living in all other household types compared with the two-adult household type (Table 2). The odds of a sole occupant being vaccinated were lower than for individuals living in a two-adult household type but higher than for individuals in all other household types. In general, lower odds ratios for vaccination were generally associated with large household types and/or household types with smaller total populations.

These findings were consistent with results from multivariable regression, which included gender, age group, health board, rural/urban location, ethnic group, and deprivation quintile as covariates (Table 3). In this multivariable analysis, the size of the effects of household type on vaccination were generally around half those in the univariable regression, with three exceptions. Firstly, the single-person household type (1) had a vaccination odds ratio of 0.70 (0.69–0.71) in both univariable and multivariable analyses. Secondly, the difference for the multi-generational adult group household type (6) was less than a factor of two, with a univariable OR of 0.62 (0.61–0.62) and a multivariable OR of 0.89 (0.88–0.90). Thirdly, the difference for the single-generation, large, adult group household type (7) was greater than a factor of two, with a univariable OR of 0.14 (0.13–0.14) and a multivariable OR of 0.52 (0.49–0.55). In addition to the effect of household type, the odds of being vaccinated were lower in younger age groups, minority ethnic background groups, if residing in a more deprived or urban area, if male, or if resident in certain health boards in the multivariable analysis (Table 3).

A multivariable regression was also conducted with the exclusion of household type as a variable to examine the impact of accounting for household composition on the odds of vaccination associated with other variables (Supplementary Table S1). Significant differences between adjusted odds ratios produced in multivariable analysis with and without the inclusion of the household type variable were seen for the 18–29 and 30–49 age groups, one health board of residence, and ethnic group variables. Compared to the White ethnic group, the adjusted odds ratio for the Asian ethnic group was 0.66 (0.64–0.67) without the inclusion of household type and 0.75 (0.73–0.77) with the inclusion of household type. In addition, the adjusted odds ratio for the Black ethnic group was 0.35 (0.33–0.36) without the inclusion of household type and 0.39 (0.37–0.40) with the inclusion of household type. Adjusted odds ratios for Other and Unknown ethnic group categories were also significantly lower in the multivariable regression which did not include household type.

## 4. Discussion

In the present study it was observed that, compared to two-adult households, all other household types were associated with a lower probability of vaccination. With the exception of the single occupancy household type (which was consistent between analyses) odds ratios from multivariable regression were less significant than those from univariable regression, indicating that in most cases, the relationship between household type and vaccination is partly mediated by other factors such as age, gender, and deprivation. In general, the lowest probabilities of vaccination were observed for the large (6–9 people) and/or uncommon household types.

Previous studies examining vaccine uptake and household composition have generally focused on household size, particularly with consideration to solitary housing. A meta-analysis by Jain et al. in 2017 [15] included an assessment of living alone as a variable for the uptake of multiple vaccines by older people in European countries and found that whilst individual studies had shown mixed results, overall living alone was associated with decreased vaccine uptake. This is consistent with the results observed here. Further studies examining influenza and zoster vaccination have also found a negative association between living alone and vaccine uptake [16,17].

Research examining inequalities of vaccine uptake based on ethnic groups has identified behavioural drivers, such as distrust of information sources, but has also revealed structural barriers [18,19]. In Wales, people of Black, Asian, or Minority ethnic background are more likely to live in an overcrowded households and this has been linked to worse outcomes for COVID-19 in these groups [20,21]. In the present study, household composition was seen to impact differences in vaccine uptake based on ethnic group and Asian, Black, and Other ethnic groups were associated with a relatively high number of unvaccinated people in large adults/children households. Although our analysis accounted for the impact of deprivation on uptake on the basis of a residential area, households are also likely to vary in level of deprivation within a residential area. Therefore, the relationships between vaccine uptake, ethnic group, large household size, and overcrowding could reflect an underlying pattern of deprivation between households.

The approach of using linkage with a residential identifier for measuring household composition has some limitations compared to other methods such as surveys or census data, particularly in relation to the level of detail. However, as this approach enables the ascertainment of full residential information at an adjustable point in time it is useful for examining variation in vaccination uptake, where phases of an immunisation programme determine the relevant time period. As household composition and vaccine uptake has not been extensively studied previously, this study may provide useful information for other countries wanting to develop vaccine equality studies.

Consideration of people living alone is of particular importance as solitary living confers a vulnerability due to social isolation [4]. In our study, living alone was associated with a decreased likelihood of vaccination compared to two-adult households. A recommendation to improve uptake for those living alone could be for vaccination programmes and primary care systems to routinely assess uptake for people who live in sole occupancy households to ensure the protection of this group. However, the probability of vaccination if living alone was not as low as in large household types. Our study also indicates that housing is a factor for differences in vaccination related to ethnic background and implicates family households in these inequalities. Although such findings could reflect an underlying pattern of deprivation, policies to improve uptake based on living in large and/or family households may be effective in reducing inequalities for these groups. These include enabling joint appointing for cohabitees where appropriate and allowing clinic opening times to accommodate those with care duties.

The findings from this study should be taken into consideration when planning the rollout of further COVID-19 booster vaccinations to avoid further widening of inequalities. Moreover, the findings from this study may be relevant to other vaccination programmes in Wales as well as across the UK and other developed countries. Further mixed-methods research should explore the causal mechanisms of differences in vaccine uptake between different types of households and whether these mechanisms are structural in nature or relate predominantly to behaviour and interpersonal relationships.

## Figures and Tables

**Table 1:** Description of household categories used to assess COVID-19 vaccine uptake.

Household Type	Definition	Relevant Housing Classifications
1. Single occupancy	Size 1	Lone dwelling.
2. Two adults	Size 2, no <18	Partnership. Cohabitation.
3. Adults/children	Size 2–5, <18 present	Family ^a^
4. Large adults/children	Size 6–9, <18 present	Large family ^a^
5. Adult group, single generation	Size 3–5, no <18, age difference <19 years	Communal residence.
6. Adult group, multiple generations	Size 3–5, no <18, age difference >18 years	Family with adult children. Communal residence.
7. Large adult group, single generation	Size 6–9, no <18, 1 generation, age difference <19 years	Large communal residence.
8. Large adult group, multiple generations	Size 6–9, no <18, >1 generation, age difference >18 years	Large family with adult children. Large communal residence.

^a^ Adults/children and large adults/children households may also include a low proportion of communal residences.

**Table 2:** Uptake of two doses of COVID-19 vaccine (any type) by household type and odds of being vaccinated, Wales ^a^.

Household Type	Population (*n*)	Vaccinated (*n*)	Uptake (%)	OR (95% CI)
1. Single occupancy	327,629	286,963	87.6%	0.70 (0.69–0.71)
2. Two adults	639,882	582,346	91.0%	Baseline
3. Adults/children	549,758	452,582	82.3%	0.46 (0.46–0.47)
4. Large adults/children	135,043	94,799	70.2%	0.23 (0.23–0.24)
5. Adult group, single generation	48,892	33,993	69.5%	0.23 (0.22–0.23)
6. Adult group, multiple generations	577,057	497,185	86.2%	0.62 (0.61–0.62)
7. Large adult group, single generation	6553	3806	58.1%	0.14 (0.13–0.14)
8. Large adult group, multiple generations	32,461	22,472	69.2%	0.22 (0.22–0.23)

^a^ Data from the Wales Immunisation System (WIS) as of 1 January 2022.

**Table 3:** Multivariable regression model estimates for vaccinations with two doses of COVID-19 vaccine (any type) Wales ^a,b^.

Variable	Category	Population (*n*)	Vaccinated (*n*)	aOR (95% CI)
Household Type	1. Single occupancy	327,629	286,963	0.70 (0.69–0.71)
	2. Two adults	639,882	582,346	Baseline
	3. Adults/children	549,758	452,582	0.83 (0.82–0.84)
	4. Large adults/children	135,043	94,799	0.48 (0.47–0.49)
	5. Adult group, single generation	48,892	33,993	0.53 (0.52–0.54)
	6. Adult group, multiple generations	577,057	497,185	0.89 (0.88–0.90)
	7. Large adult group, single generation	6553	3806	0.52 (0.49–0.55)
	8. Large adult group, multiple generations	32,461	22,472	0.45 (0.43–0.46)
Gender	Female	1,161,191	1,016,737	Baseline
	Male	1,156,084	957,409	0.73 (0.72–0.74)
Age Group	18–29	402,223	298,465	0.30 (0.30–0.30)
	30–49	731,813	575,472	0.39 (0.39–0.40)
	50–69	760,289	694,063	Baseline
	70+	422,950	406,146	2.05 (2.01–2.08)
Health Board	Health Board 1	438,803	380,953	Baseline
	Health Board 2	502,623	427,760	0.72 (0.71–0.73)
	Health Board 3	369,837	303,933	0.86 (0.85–0.88)
	Health Board 4	336,450	294,497	1.04 (1.03–1.06)
	Health Board 5	281,646	241,400	0.73 (0.72–0.74)
	Health Board 6	94,642	81,317	0.67 (0.65–0.68)
	Health Board 7	293,274	244,286	0.74 (0.73–0.75)
Location Classification	Rural	712,806	626,082	1.15 (1.14–1.16)
	Urban	1,604,469	1,348,064	Baseline
Ethnic Group	Asian	44,767	34,775	0.75 (0.73–0.77)
	Black	11,773	7475	0.39 (0.37–0.40)
	Mixed	31,523	21,598	0.46 (0.45–0.47)
	Other	10,432	6740	0.40 (0.39–0.42)
	Unknown	235,552	158,880	0.33 (0.32–0.33)
	White	1,983,228	1,744,678	Baseline
Deprivation Quintile	1 (Most deprived)	452,962	356,673	0.47 (0.46–0.47)
	2	461,071	386,911	0.60 (0.59–0.61)
	3	468,108	397,891	0.68 (0.67–0.69)
	4	465,345	409,365	0.80 (0.79–0.81)
	5 (Least deprived)	469,789	423,306	Baseline

^a^ Data from the Wales Immunisation System as of 1 January 2022. ^b^ Deprivation quintile was calculated using Lower-layer Super Output Areas of residence, ranked according to the Welsh Index of Multiple Deprivation.

## Data Availability

The data used in this study is available from the SAIL Databank at Swansea University, Swansea, UK, which is part of the national e-health records research infrastructure for Wales. SAIL has established an application process to be followed by anyone who would like to access data via SAIL at: www.saildatabank.com/application-process.

## Supplementary Materials

The following supporting information can be downloaded at: https://www.mdpi.com/article/10.3390/vaccines11030604/s1, Table S1: Multivariate regression model estimates for vaccination with two doses of COVID-19 vaccine (any type), not including household type as a variable, Wales; Table S2: Study population by household type, number of people stratified for sex, age group and Asian, Black, Mixed and Other ethnic group categories, Wales 2020–2021; Table S3: Study population by household type, percentage of people stratified for sex, age group and Asian, Black, Mixed and Other ethnic group categories, Wales 2020–2021.
