# Supplementary material for: Household Composition and Inequalities in COVID-19 Vaccination in Wales, UK
Source: Vaccines (Basel). 2023 Mar 7;11(3):604. doi: 10.3390/vaccines11030604 (PMC10055803; doi:10.3390/vaccines11030604)
Supplement: Supplementary file 1 [file vaccines-11-00604-s001.zip › vaccines-2219068-supplementary.pdf]

Supplementary Table S1. Multivariate regression model estimates for vaccination with two doses of COVID-19 vaccine (any type), not including household type as a variable, Wales.[a,b]

Variable	Category	Population (n)	Vaccinated (n)	aOR (95% CI)
Gender	Female	1,161,191	1,016,737	*
	Male	1,156,084	957,409	0.73 (0.73-0.74)
Age Group	18-29	402,223	298,465	0.28 (0.28-0.28)
	30-49	731,813	575,472	0.37 (0.37-0.37)
	50-69	760,289	694,063	*
	70+	422,950	406,146	2.05 (2.01-2.09)
Health Board	Health Board 1	438,803	380,953	*
	Health Board 2	502,623	427,760	0.71 (0.70-0.72)
	Health Board 3	369,837	303,933	0.83 (0.82-0.84)
	Health Board 4	336,450	294,497	1.04 (1.03-1.06)
	Health Board 5	281,646	241,400	0.72 (0.71-0.73)
	Health Board 6	94,642	81,317	0.66 (0.65-0.68)
	Health Board 7	293,274	244,286	0.73 (0.72-0.74)
Location Classification	Rural	712,806	626,082	1.16 (1.15-1.17)
	Urban	1,604,469	1,348,064	*
Ethnicity	Asian	44,767	34,775	0.66 (0.64-0.67)
	Black	11,773	7,475	0.35 (0.33-0.36)
	Mixed	31,523	21,598	0.44 (0.43-0.46)
	Other	10,432	6,740	0.36 (0.35-0.38)
	Unknown	235,552	158,880	0.31 (0.31-0.31)
	White	1,983,228	1,744,678	*
Deprivation Quintile	1	452,962	356,673	0.45 (0.45-0.46)
	2	461,071	386,911	0.59 (0.58-0.59)
	3	468,108	397,891	0.66 (0.65-0.67)
	4	465,345	409,365	0.79 (0.78-0.80)
	5	469,789	423,306	*

[a] Data from the Wales Immunisation System as at 1st January 2022.

[b] Deprivation quintile was calculated using Lower-layer Super Output Areas of residence, ranked according to the Welsh Index of Multiple Deprivation.

Supplementary Table S2. Study population by household type, number of people stratified for sex, age group and Asian, Black, Mixed and Other ethnic group categories, Wales 2020-21.

Stratification	Household Type							
	1	2	3	4	5	6	7	8
None (all)	327,629	639,882	549,758	135,043	48,892	577,057	6,553	32,461
Male	152,625	322,776	241,270	69,221	27,866	319,466	3,682	19,178
Female	175,004	317,106	308,488	65,822	21,026	257,591	2,871	13,283
18-29	22,772	51,272	103,767	37,639	16,660	154,561	4,492	11,060
30-49	63,964	103,864	343,382	66,208	16,295	127,686	1,589	8,825
50-69	112,016	271,654	93,893	25,609	12,158	235,672	214	9,073
70+	128,877	213,092	8,716	5,587	3,779	59,138	258	3,503
Asian	2,417	5,414	13,476	9,764	1,731	9,488	482	1,995
Black	1,206	1,613	3,302	2,269	541	2,085	165	592
Mixed	3,470	5,190	10,077	3,893	986	6,930	180	797
Other	807	1,404	3,108	2,191	533	1,876	114	399

Supplementary Table S3. Study population by household type, percentage of people stratified for sex, age group and Asian, Black, Mixed and Other ethnic group categories, Wales 2020-21.

Stratification	Household Type							
	1	2	3	4	5	6	7	8
None (all)	14.1%	27.6%	23.7%	5.8%	2.1%	24.9%	0.3%	1.4%
Male	13.2%	27.9%	20.9%	6.0%	2.4%	27.6%	0.3%	1.7%
Female	15.1%	27.3%	26.6%	5.7%	1.8%	22.2%	0.2%	1.1%
18-29	5.7%	12.7%	25.8%	9.4%	4.1%	38.4%	1.1%	2.7%
30-49	8.7%	14.2%	46.9%	9.0%	2.2%	17.4%	0.2%	1.2%
50-69	14.7%	35.7%	12.3%	3.4%	1.6%	31.0%	0.0%	1.2%
70+	30.5%	50.4%	2.1%	1.3%	0.9%	14.0%	0.1%	0.8%
Asian	5.4%	12.1%	30.1%	21.8%	3.9%	21.2%	1.1%	4.5%
Black	10.2%	13.7%	28.0%	19.3%	4.6%	17.7%	1.4%	5.0%
Mixed	11.0%	16.5%	32.0%	12.3%	3.1%	22.0%	0.6%	2.5%
Other	7.7%	13.5%	29.8%	21.0%	5.1%	18.0%	1.1%	3.8%
